# Circulating microRNA molecular signatures converge with erythroid phenotypes and iron homeostasis in pediatric tic disorders

**DOI:** 10.3389/fpsyt.2026.1807959

**Published:** 2026-05-04

**Authors:** Ru Jia, Tingting Zhu, Xue Tian, Simeng Wang, Si Zhang, Fei Fan, Yaru Wang, Yuxin Chai, Zilin Chen, Yuchen Hu, Weifeng Li, Fei Han

**Affiliations:** 1 Department of Pediatric, Guang’anmen Hospital, China Academy of Chinese Medical Sciences, Beijing, China; 2 Department of Cardiovascular, Guang’anmen Hospital, China Academy of Chinese Medical Sciences, Beijing, China; 3 Department of Pediatric, Suzhou Hospital of Traditional Chinese Medicine, Suzhou, Jiangsu, China

**Keywords:** biomarker integration, circulating microRNAs, erythroid phenotypes, iron metabolism, pediatric, tic disorders

## Abstract

**Introduction:**

Tic disorders (TDs) are common neurodevelopmental conditions with unclear pathogenesis and a lack of objective biomarkers. This study aimed to explore the associations among circulating microRNAs (miRNAs), erythroid phenotypes, iron homeostasis, and pediatric TD.

**Methods:**

A total of 30 TD children and 10 healthy controls were enrolled. Serum levels of five candidate miRNAs, erythroid parameters, and iron metabolism indicators were detected. In this study, serum levels of five candidate miRNAs were quantified in children with tic disorders and age- and sex-matched normal controls using RT-qPCR. Erythroid phenotypes and serum iron-metabolism indicators were assessed in parallel. Differential expression analyses, multivariate modeling, and network-based target and functional enrichment analyses were performed to explore integrated molecular signatures.

**Results:**

Results showed that hsa-miR-125b-5p and hsa-miR-23a-3p were significantly upregulated in the TD group. TD children exhibited lower hemoglobin, mean corpuscular volume (MCV), serum ferritin, transferrin (TrF), total ironbinding capacity, and soluble transferrin receptor, along with higher mean corpuscular hemoglobin concentration, while hemoglobin fractions remained unchanged. The integrated model combining hsa-miR-125b-5p, MCV, and TrF showed excellent diagnostic performance (AUC=0.977). Network and enrichment analyses revealed convergent biological pathways involving cellular and multicellular homeostasis, metal ion regulation, and TGF-β/BMP-associated signaling, linking miRNA-associated regulatory networks to erythroid and ironrelated processes.

**Discussion:**

In conclusion, children with TD may exhibit homeostatic dysregulation of circulating miRNAs, erythroid profiles, and iron metabolism. This integrative molecular framework may provide insight into peripheral regulatory mechanisms relevant to neurodevelopmental pathology.

## Introduction

Tic disorders (TDs) are childhood-onset neurodevelopmental and neuropsychiatric conditions characterized by recurrent motor and/or vocal tics with a typically fluctuating course. Clinically, TDs are classified according to tic type and duration into provisional tic disorder (PTD), persistent (chronic) motor or vocal tic disorder (CTD), Tourette syndrome (TS), and other or unspecified tic disorders, as defined by the *Diagnostic and Statistical Manual of Mental Disorders, Fifth Edition, Text Revision* (DSM-5-TR) (1). TDs frequently co-occur with attention-deficit/hyperactivity disorder (ADHD), obsessive-compulsive disorder (OCD) or obsessive-compulsive symptoms, and internalizing symptoms such as anxiety and depression, which substantially increase functional impairment and reduce quality of life (2). At present, TDs, including Tourette syndrome, are diagnosed largely on the basis of clinical phenomenology and history, and the process is complex and time-consuming. Despite advances in understanding the genetic architecture, neurocircuitry, and immune-metabolic contributions to TD pathomechanism, validated objective molecular markers that can robustly reflect underlying biological heterogeneity and disease-associated regulatory processes remain unavailable (3). Systematic evaluations of peripheral biomarkers in TS have identified multiple candidate signals (4). However, these studies have also highlighted pronounced heterogeneity, limited reproducibility, and the need for larger, standardized investigations before clinical translation can be achieved.

Circulating microRNAs (miRNAs) are small non-coding RNAs that regulate gene expression at the post-transcriptional level. Evidence indicates that circulating miRNAs exhibit relative stability in biofluids and hold promise as potential biomarkers for clinical disease diagnosis (5–7). Their compatibility with standardized reverse transcription-quantitative polymerase chain reaction (RT-qPCR) workflows and their capacity to reflect system-level regulatory programs further support their application as liquid-biopsy biomarkers across a wide range of diseases (8–10). Beyond their technical advantages, miRNAs are key post-transcriptional regulators of multiple biological processes relevant to brain disorders, including neurodevelopment, synaptic plasticity, immune signaling, and stress responses. Accordingly, miRNA dysregulation has been implicated across numerous neuropsychiatric and neurodevelopmental conditions (11–14). In pediatric TD, some studies have reported disease-associated circulating miRNA signals. Reduced serum miR-429 levels have been described in TS (15). Plasma small extracellular vesicle-derived miRNA panels have been proposed as potential biomarkers for TD, such as members of the hsa-let-7 family, hsa-miR-125b-5p, and so on (16). These findings suggest that circulating miRNAs may capture peripheral molecular correlates of TD biology, supporting the feasibility of miRNA-based diagnostic approaches.

Beyond neuropsychiatric comorbidities, peripheral physiology potentially relevant to TD has been increasingly considered. Reduced ferritin has been reported in pediatric TD cohorts, and lower ferritin has been associated with greater tic severity in children with TS (17, 18). Convergent imaging-transcriptomic evidence has further suggested disturbed iron homeostasis in Gilles de la Tourette syndrome, motivating integrated assessment of iron-related phenotypes alongside molecular markers (19). Iron metabolism is tightly coupled to erythropoiesis and hemoglobin synthesis, as erythroid precursors consume most circulating iron for hemoglobin production and iron availability constrains red-cell production. Reviews of iron-status assessment emphasize the interpretability of routine biomarkers, such as serum ferritin (iron stores), transferrin-related measures (transport capacity), and soluble transferrin receptor (functional iron demand), when contextualizing erythroid/hemoglobin phenotypes in blood (20–22). In addition, occasional observations have suggested possible intersections between TD and erythroid/hemoglobin-related biology (23). A hypothesis has been proposed that mild erythroid phenotypes or hemoglobin alterations may disrupt neuronal oxygenation and antioxidant defense by affecting oxidative metabolism, while the globin gene is located near the genetic locus of psychiatric disorders (23). Therefore, erythroid phenotypes or hemoglobin alterations may be involved in the pathogenesis of psychiatric diseases. Although the evidence has not established causality and requires cautious interpretation, these observations have provided motivation to study erythroid/hemoglobin-related phenotypes in TD cohorts. Importantly, miRNA nodes implicated in TD also intersect with erythroid/hemoglobin biology. For instance, miR-23a and miR-144 have been reported to be potentially associated with TS (24). In parallel, miR-144 and miR-23a/27a have established regulatory roles in globin gene expression and erythropoiesis (25, 26), and a conserved miR-144/Hmgn2 axis has been shown to orchestrate chromatin organization during erythropoiesis (27). Together, these findings provide a theoretical basis for investigating cross-domain phenotypes linking TD with hematological features and iron metabolism indicators.

Therefore, a miRNA-centered case-control study was conducted in children with TD, in which candidate miRNAs implicated in both TD and hemoglobin/erythropoiesis-related contexts were prioritized for serum validation. Erythroid/hemoglobin phenotypes and iron metabolism indicators were measured in parallel to test whether an integrated peripheral signature could be delineated and whether multi-domain integration could improve separation of TD-associated molecular profiles from those of controls.

## Results

### Demographic characteristics

Peripheral blood samples were obtained from 30 children with tic disorders (TD group) and 10 age-matched and sex-matched normal controls (NC group). Children in the TD group ranged from 5 to 17 years of age (mean ± SD: 8.93 ± 2.80 years), with a male-to-female ratio of 24:6. Among the TD patients, 9 were diagnosed with PTD, 8 with CTD, and 13 with TS. The mean duration of illness in the TD group was 2.25 ± 1.86 years, and the mean Yale Global Tic Severity Scale (YGTSS) score was 27.97 ± 10.59. The NC group ranged from 7 to 18 years of age (mean ± SD: 10.25 ± 3.16 years), with a male-to-female ratio of 8:2. All participants were recruited and clinically evaluated at the Department of Pediatrics, Guang’anmen Hospital, China Academy of Chinese Medical Sciences. No significant differences were observed between the TD and NC groups with respect to age (*p* = 0.286) or sex distribution (*p* = 1.000). A summary of the demographic characteristics of the study participants is provided in **Table 1**. Detailed data and additional analyses are provided in **Supplementary Material ["Additional file 1"]**.

**Table 1:** Demographic and clinical characteristics.

Characteristics	TD	NC	*p*-value
Mean age (± SD)	8.93 (± 2.80)	10.25 (± 3.16)	0.286
Sex (M:F)	24:6	8:2	1.000
Subtypes of TD (PTD: CTD : TS)	9:8:13		
YGTSS	27.97 (± 10.59)		
Duration of illness (years)	2.25 (± 1.86)		
Sample size	30	10	

TD, Tic Disorder; NC, Normal Controls; SD, Standard Deviation; M, Male; F, Female; PTD, Provisional tic disorder; CTD, Chronic tic disorder; TS, Tourette syndrome.

### Selection of candidate circulating miRNAs

**Figure 1:**
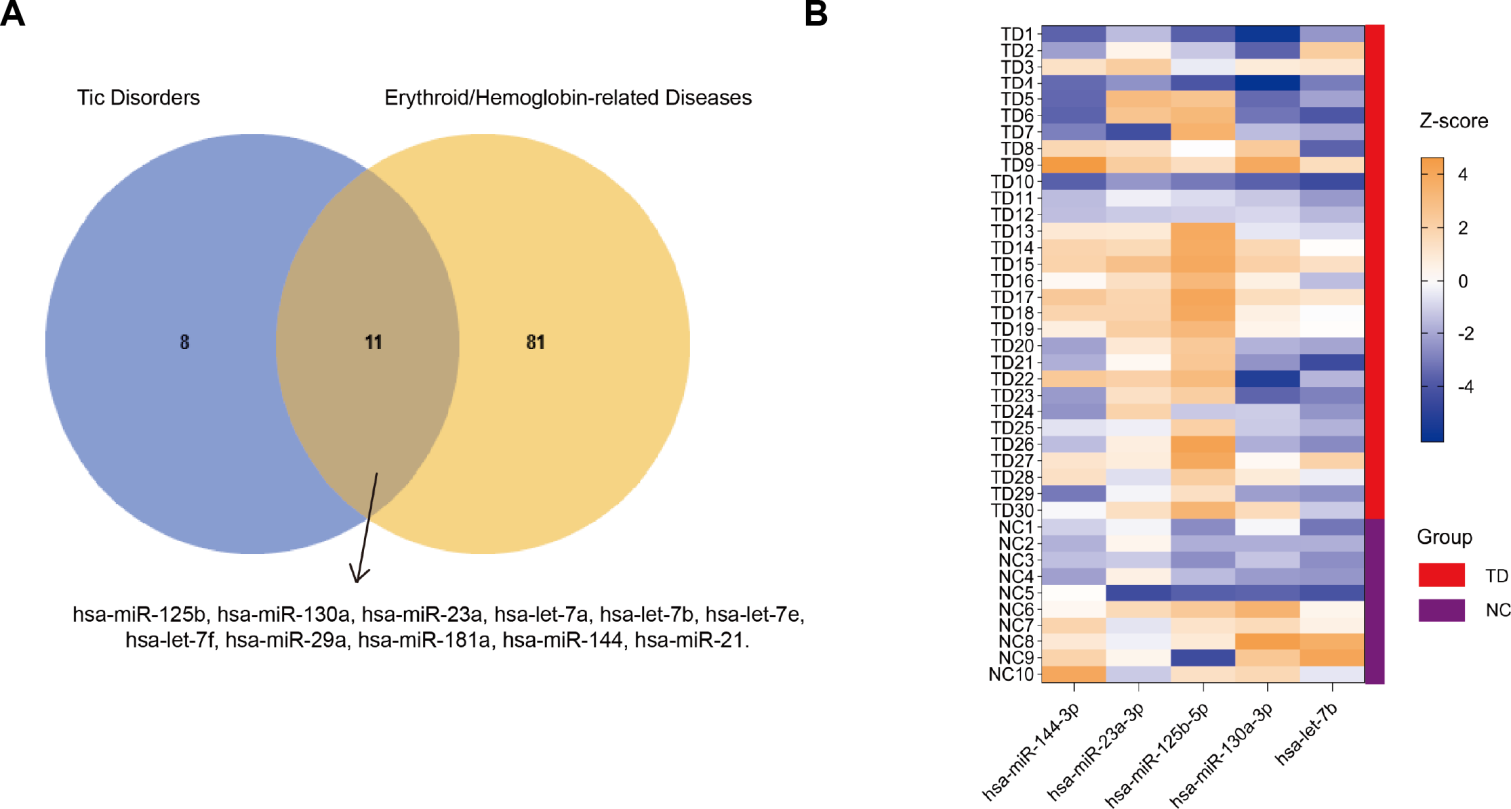
Selection and expression of candidate circulating miRNAs. **(A)** Venn diagram showing the overlap between miRNAs associated with TDs and those associated with erythroid/hemoglobin-related diseases. Eleven miRNAs were shared between the two categories. **(B)** Heatmap showing normalized serum expression levels of selected candidate miRNAs in TD and NC groups. The color scale indicates *Z*-score-standardized log base 2 fold change (log_2_FC), with warmer colors representing higher expression levels and cooler colors representing lower expression levels. The annotation bar indicates group membership, with the TD group shown in red, and the NC group shown in purple.

Candidate miRNAs were selected using a stepwise screening strategy that integrated TD-related and erythroid/hemoglobin-related disease contexts. Using the Human microRNA Disease Database version 4.0 (HMDD v4.0, http://www.cuilab.cn/hmdd) (28), miRNAs associated with TD or erythroid/hemoglobin-related diseases were retrieved. In addition, certain miRNAs associated with diseases were supplemented from Gene Expression Omnibus (GEO, https://www.ncbi.nlm.nih.gov/geo/) and published literature (15, 24, 29, 30). Detailed data and additional analyses are provided in **Supplementary Material ["Additional file 2"]**. Overlap analysis identified 11 miRNAs shared between the two disease categories (**Figure 1A**). These overlapping miRNAs were prioritized as candidate circulating miRNAs for subsequent experimental validation. Based on previous reports, the availability of well-defined mature miRNA sequences, and assay feasibility considerations, five miRNAs were selected for further expression analysis, including hsa-miR-23a-3p, hsa-miR-125b-5p, hsa-miR-130a-3p, hsa-miR-144-3p, and hsa-let-7b (15, 24).

### Circulating miRNA expression

Standardized miRNA expression profiles demonstrated a tendency toward separation between the TD and NC groups, with hsa-miR-125b-5p contributing most prominently to this pattern (**Figure 1B**). Independent-samples statistical tests were subsequently performed for each of the five candidate miRNAs. Detailed data and additional analyses are provided in **Supplementary Material ["Additional file 1"]**.

**Figure 2:**
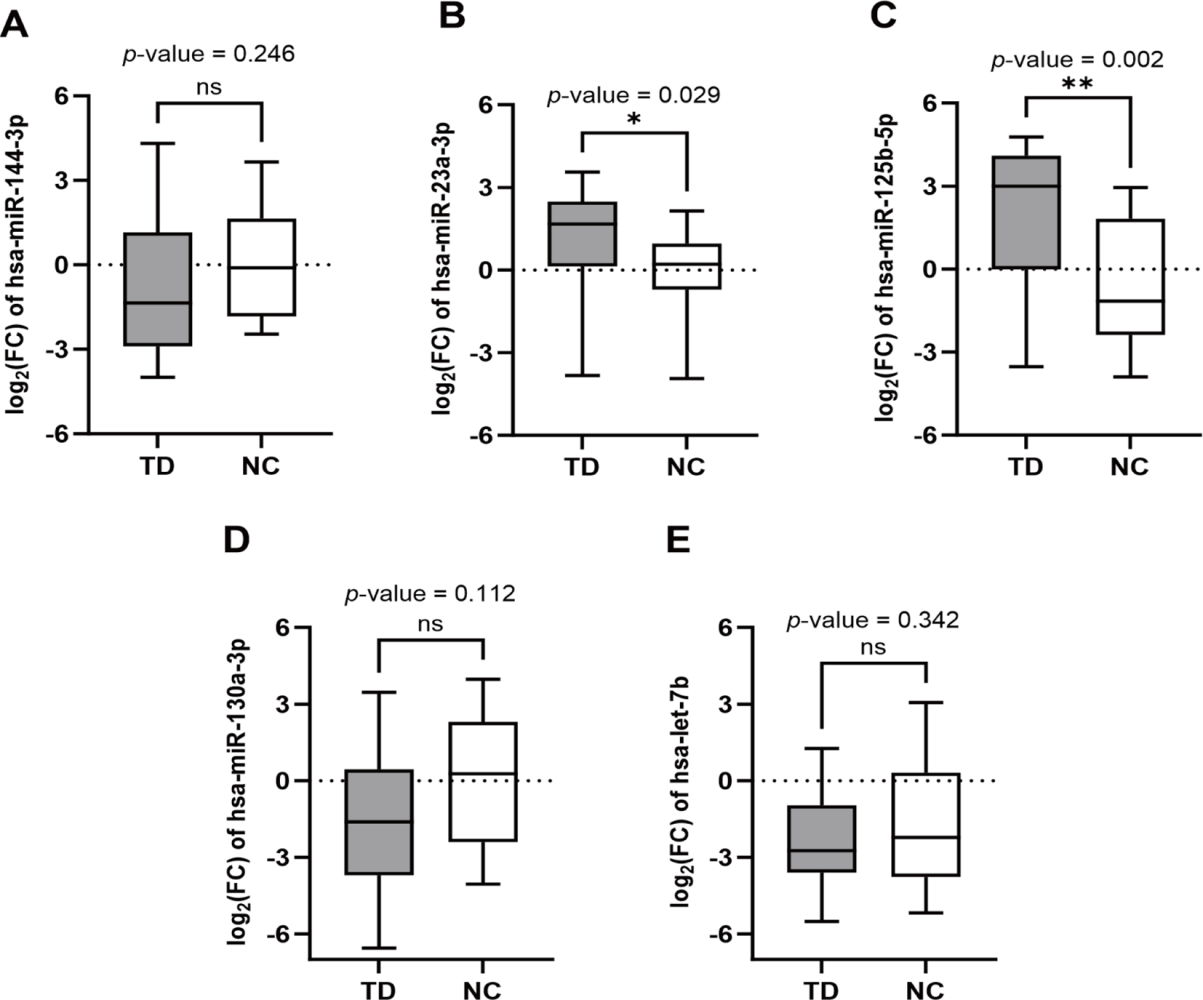
Differential expression of candidate circulating miRNAs. Boxplots showing serum expression levels of five candidate miRNAs measured by RT-qPCR in children with TD and normal controls (NC). Expression values are presented as log_2_(FC) relative to the NC group, calculated using the 2^−ΔΔCt^ method with U6 as the endogenous reference. **(A)** hsa-miR-144-3p (*Mann-Whitney U* test, *p* = 0.246). **(B)** hsa-miR-23a-3p (*Mann-Whitney U* test, *p* = 0.029). **(C)** hsa-miR-125b-5p (*Mann-Whitney U* test, *p* = 0.002). **(D)** hsa-miR-130a-3p (*t-*test, *p* = 0.112). **(E)** hsa-let-7b (*t-*test, *p* = 0.342). "*" means *p*-value < 0.05, "**" means *p*-value < 0.01, "ns" means *p*-value > 0.05.

Among the five candidate miRNAs, hsa-miR-23a-3p (*Mann-Whitney U* test, *p* = 0.029; **Figure 2B**) and hsa-miR-125b-5p (*Mann-Whitney U* test, *p* = 0.002; **Figure 2C**) were significantly upregulated in the TD group compared with the NC group. In contrast, no statistically significant differences were detected for hsa-miR-144-3p, hsa-miR-130a-3p, or hsa-let-7b between groups (**Figures 2A, D, E**). Although not all comparisons reached statistical significance, consistent directional patterns were observed. Compared with the NC group, hsa-miR-144-3p, hsa-miR-130a-3p, and hsa-let-7b tended to show a slight down-regulation trend in the TD group (**Figures 2A, D, E**).

### Erythroid and hemoglobin-related phenotypes

High resolution analysis of hemoglobin components revealed several differences between the TD and NC groups (**Table 2**). Detailed data and additional analyses are provided in **Supplementary Material ["Additional file 1"]**. Among erythroid phenotype-related parameters, hemoglobin concentration (Hb) was lower in the TD group than in the NC group (*p* = 0.046), although values remained within the physiologic reference range. Mean corpuscular volume (MCV) was significantly reduced in the TD group (*p* = 0.008). Conversely, mean corpuscular hemoglobin concentration (MCHC) was increased in the TD group (*p* = 0.043). No significant differences were observed for red blood cell count (RBC) or mean corpuscular hemoglobin (MCH) between groups. Regarding hemoglobin fractions, no significant differences were detected for hemoglobin A (HbA), hemoglobin A_2_ (HbA_2_), or fetal hemoglobin (HbF) between groups.

**Table 2:** High resolution analysis of hemoglobin components and serum iron metabolism indicators results.

Indicators	TD	NC	*p*-value
^1^RBC (×10^12^/L)	4.86 (± 0.44)	5.01 (± 0.32)	0.335
^2^Hb (g/L)	133.77 (± 11.30)	141.10 (± 11.47)	0.046^*^
^2^MCV (fl)	84.54 (± 5.28)	89.02 (± 4.56)	0.008^**^
^2^MCH (pg)	27.60 (± 1.76)	28.15 (± 1.22)	0.488
^2^MCHC (g/L)	326.37 (± 9.44)	316.70 (± 13.26)	0.043^*^
^2^HbA (%)	96.89 (± 0.57)	96.98 (± 0.56)	0.747
^2^HbA_2_ (%)	2.84 (± 0.21)	2.72 (± 0.51)	0.866
^2^HbF (%)	0.26 (± 0.49)	0.30 (± 0.49)	0.890
^2^SI (μmol/L)	17.02 (± 5.94)	18.70 (± 8.50)	0.963
^1^SF (μg/L)	49.13 (± 23.19)	70.13 (± 30.44)	0.028^*^
^2^TrF (mg/dl)	224.64 (± 24.50)	277.67 (± 45.04)	0.001^**^
^2^sTfR (nmol/L)	36.36 (± 8.83)	43.22 (± 9.68)	0.028^*^
^2^TIBC (μmol/L)	64.18 (± 7.98)	75.76 (± 10.81)	0.002^**^

Data are presented as mean ± standard deviation (SD).

^1^The indicators were compared by using independent-samples *t*-test.

^2^The indicators were compared by using independent-samples *Mann-Whitney U* test.

^*^p-value < 0.05.

^**^p-value < 0.01.

RBC, Red Blood Cell Count; Hb, Hemoglobin; MCV, Mean Corpuscular Volume; MCH, Mean Corpuscular Hemoglobin; MCHC, Mean Corpuscular Hemoglobin Concentration; HbA, Hemoglobin A; HbA_2_, Hemoglobin A_2_; HbF, Fetal Hemoglobin; SI, Serum Iron; SF, Serum Ferritin; TrF, Transferrin; sTfR, Soluble Transferrin Receptor; TIBC, Total Iron-Binding Capacity.

### Serum iron metabolism indicators

Serum iron metabolism indicators were further assessed in the TD and NC groups (**Table 2**). Detailed data and additional analyses are provided in **Supplementary Material ["Additional file 1"]**. Serum ferritin (SF) levels were significantly lower in the TD group than in the NC group (*p* = 0.028). In addition, transferrin (TrF) and total iron-binding capacity (TIBC) were significantly reduced in the TD group (TrF, *p* = 0.001; TIBC, *p* = 0.002). Soluble transferrin receptor (sTfR) was also lower in the TD group (*p* = 0.028). By contrast, serum iron (SI) did not differ significantly between groups.

### Correlation analysis

**Figure 3:**
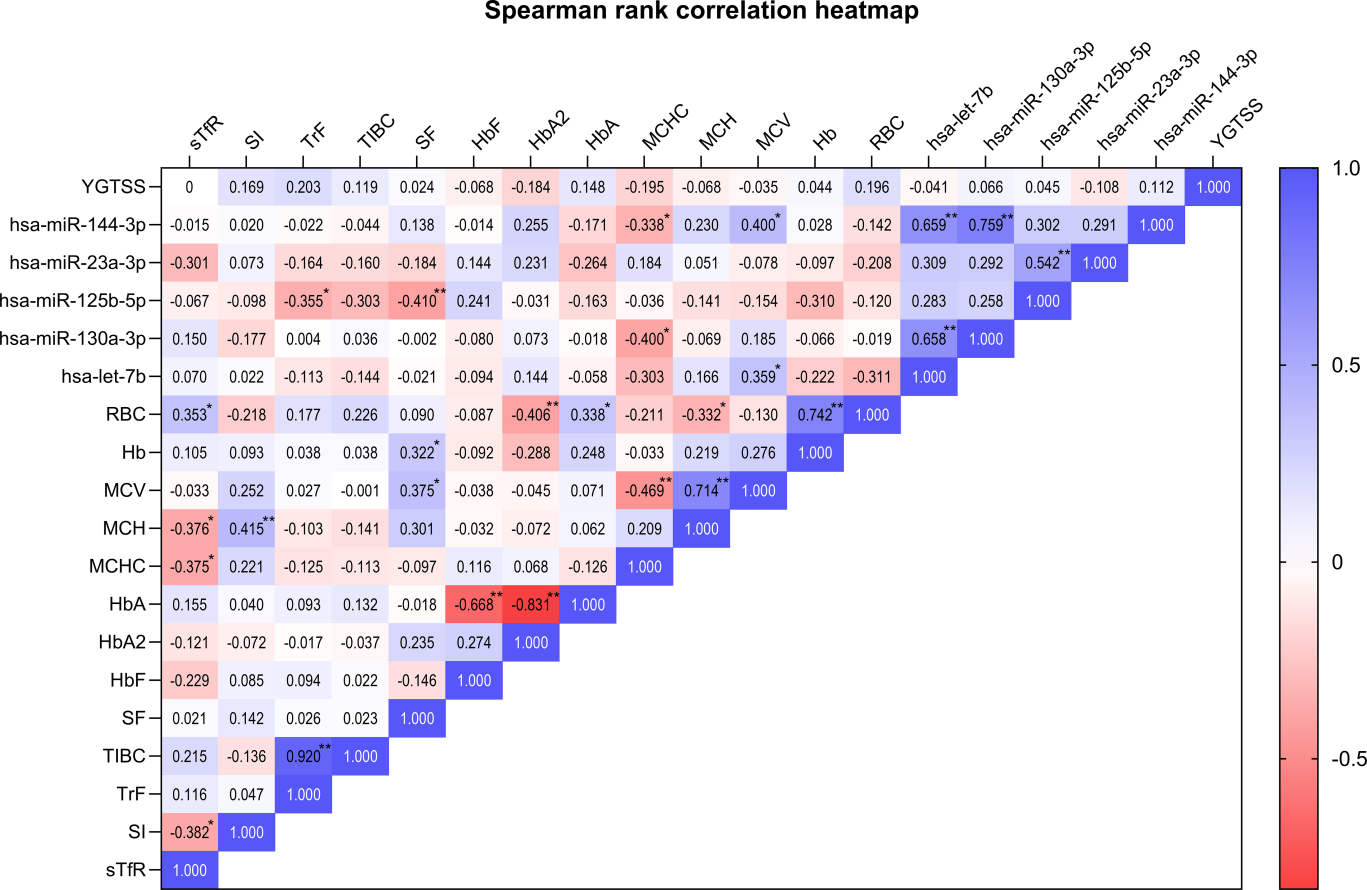
Spearman rank correlation heatmap of clinical severity, miRNA expression and hematological parameters in TD group.The color gradient from red (negative correlation) to blue (positive correlation) represents the strength of the correlation, with the numbers in the grid indicating the correlation coefficients (r). “*” means *p*-value < 0.05, “**” means *p*-value < 0.01. The expression levels of miRNAs (including hsa-miR-144-3p, hsa-miR-130a-3p, hsa-miR-125b-5p, hsa-miR-23a-3p and hsa-let-7b) used in the correlation analysis were log_2_FC values.

Spearman rank correlation analysis was performed to explore the associations among YGTSS, hematological parameters, and miRNA expression levels in children with TD (**Figure 3**). First, no significant correlation was observed between the YGTSS score and other indicators. Second, multiple significant correlations were detected among the candidate miRNAs. Specifically, hsa-miR-144-3p was significantly positively correlated with hsa-miR-130a-3p (r = 0.759, *p* < 0.001) and hsa-let-7b (r = 0.659, *p* < 0.001); hsa-miR-130a-3p was significantly positively correlated with hsa-let-7b (r = 0.658, *p* < 0.001); hsa-miR-23a-3p was significantly positively correlated with hsa-miR-125b-5p (r = 0.542, *p* < 0.001). In terms of the correlation between miRNAs and hematological indicators, hsa-miR-125b-5p was significantly negatively correlated with SF (r = −0.410, *p = *0.009) and TrF (r = −0.355, *p = *0.025), while hsa-miR-144-3p was significantly positively correlated with MCV (r = 0.400, *p = *0.011) and negatively correlated with MCHC (r = −0.338, *p = *0.033). Hsa-miR-130a-3p was significantly negatively correlated with MCHC (r = −0.400, *p = *0.010) and hsa-let-7b was significantly positively correlated with MCV (r = 0.359, *p = *0.023).

### Subgroup analysis

Subgroup comparisons were performed among children with PTD, CTD, and TS. Overall, most hematological parameters and miRNA expression levels showed no significant differences across the three TD subtypes. Only one significant overall intergroup difference was observed in the expression level of hsa-miR-125b-5p (*p* = 0.028). Multiple comparisons revealed significant differences between the PTD and CTD subgroups in serum ferritin (SF, *p* = 0.026), hsa-miR-130a-3p (*p* = 0.037), and hsa-miR-125b-5p (*p* = 0.037). The expression of hsa-miR-130a-3p was higher in the PTD group, while SF level and hsa-miR-125b-5p expression were higher in the CTD group. No significant differences were detected between the PTD and TS groups, or between the CTD and TS groups for any of the indicators.

### Integrated diagnostic modeling

To evaluate the diagnostic performance of the identified biomarkers, univariate and multivariate binary logistic regression analyses were performed (**Table 3**). Variables showing statistical significance in group comparisons (*p* < 0.05) were first entered into univariate logistic regression models. Six variables were significantly associated with TD status in univariate analyses, including hsa-miR-125b-5p, MCV, MCHC, SF, TrF, and TIBC. These six variables were subsequently entered into a multivariate logistic regression model. Stepwise variable selection using the likelihood-ratio method retained three predictors in the final model, including hsa-miR-125b-5p, MCV, and TrF. In the multivariate model, MCV (*p* = 0.035) and TrF (*p* = 0.028) remained statistically significant, whereas hsa-miR-125b-5p showed a trend toward significance (*p* = 0.079) but did not meet the predefined threshold for independent association.

**Table 3:** Binary logistic regression analysis of biomarkers associated with TD.

Indicators	Univariate logistic regression analyses			Multivariate logistic regression analyses		
	OR	95% CI	*p*-value	OR	95% CI	*p*-value
hsa-miR-125b-5p (-ΔCt)	1.485	1.094 - 2.015	0.011^*^	2.037	0.920-4.507	0.079
hsa-miR-23a-3p (-ΔCt)	1.448	0.955 - 2.196	0.081			
Hb (g/L)	0.948	0.891 - 1.010	0.097			
MCV (fl)	0.778	0.626 - 0.967	0.024^*^	0.506	0.268-0.953	0.035^*^
MCHC (g/L)	1.082	1.010 - 1.159	0.026^*^			
SF (μg/L)	0.969	0.939 - 0.999	0.041^*^			
TrF (mg/dl)	0.952	0.923 - 0.983	0.003^**^	0.916	0.848-0.990	0.028^*^
sTfR (nmol/L)	0.928	0.856 - 1.005	0.066			
TIBC (μmol/L)	0.875	0.792 - 0.966	0.008^**^			

^*^ *p*-value < 0.05.

^**^p-value < 0.01.

OR, Odds ratio; 95% CI, 95% confidence interval.

**Figure 4:**
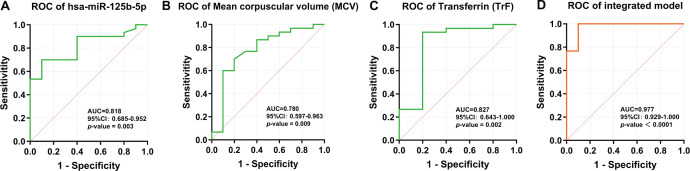
ROC curves illustrating the diagnostic performance of individual predictors and the integrated multivariate model for distinguishing children with TD from NC. **(A)** hsa-miR-125b-5p. **(B)** MCV. **(C)** TrF. **(D)** Integrated logistic regression model combining hsa-miR-125b-5p, MCV, and TrF. AUCs with corresponding confidence intervals were calculated for each curve.

Receiver operating characteristic (ROC) curve analyses were conducted for each individual predictor and for the integrated multivariate model (**Figures 4A–D**). Among the single predictors, hsa-miR-125b-5p [An area under the curve (AUC) = 0.818, a 95% confidence interval (95% CI): 0.685 to 0.952, *p* = 0.003], MCV (AUC = 0.780, 95% CI: 0.597 to 0.963, *p* = 0.009) and TrF (AUC = 0.827, 95% CI: 0.643 to 1.000, *p* = 0.002) demonstrated moderate discriminative ability according to commonly used criteria (31). The multivariate regression model, integrating hsa-miR-125b-5p, MCV, and TrF, provided the best diagnostic performance (AUC = 0.977, 95% CI: 0.929 to 1.000, *p* < 0.0001). Further analysis showed that the diagnostic model achieved 100% sensitivity and 90% specificity. The optimal cutoff for the predictive probability was determined to be 0.413 for distinguishing TD patients from NC.

To assess model robustness, five-fold cross-validation was performed, demonstrating consistent performance across folds. The mean AUC from cross-validation was 0.981 and the standard deviation was 0.006. In addition, bootstrap resampling yielded a 95% CI for the AUC ranging from 0.877 to 0.977, further supporting the stability of the integrated diagnostic model.

### Targets identification and network analysis

#### Targets retrieval and network construction

For each variable, the targets were obtained using associated gene interaction resources. The targets’ first-neighbor interacting partners were retrieved using the STRING v12.0 database (https://string-db.org/) (32), and were limited to no more than 50. Molecular interaction networks were subsequently constructed and analyzed using Cytoscape v3.8.1. Functional enrichment analysis was performed using Metascape v3.5.20250701 (https://metascape.org/) (33), in which all nodes within the network were compared against the entire genome background to identify statistically overrepresented biological functions. Enrichment analyses were conducted across multiple databases, including Gene ontology (GO), Kyoto encyclopedia of genes and genomes (KEGG), Reactome, WikiPathways, and BioCarta. More specifically, *p*-values were calculated based on the cumulative hypergeometric distribution (*p < *0.01), and *q*-values were calculated using the Benjamini-Hochberg procedure to account for multiple testing.

#### Targets network and enrichment analysis of hsa-miR-125b-5p

**Figure 5:**
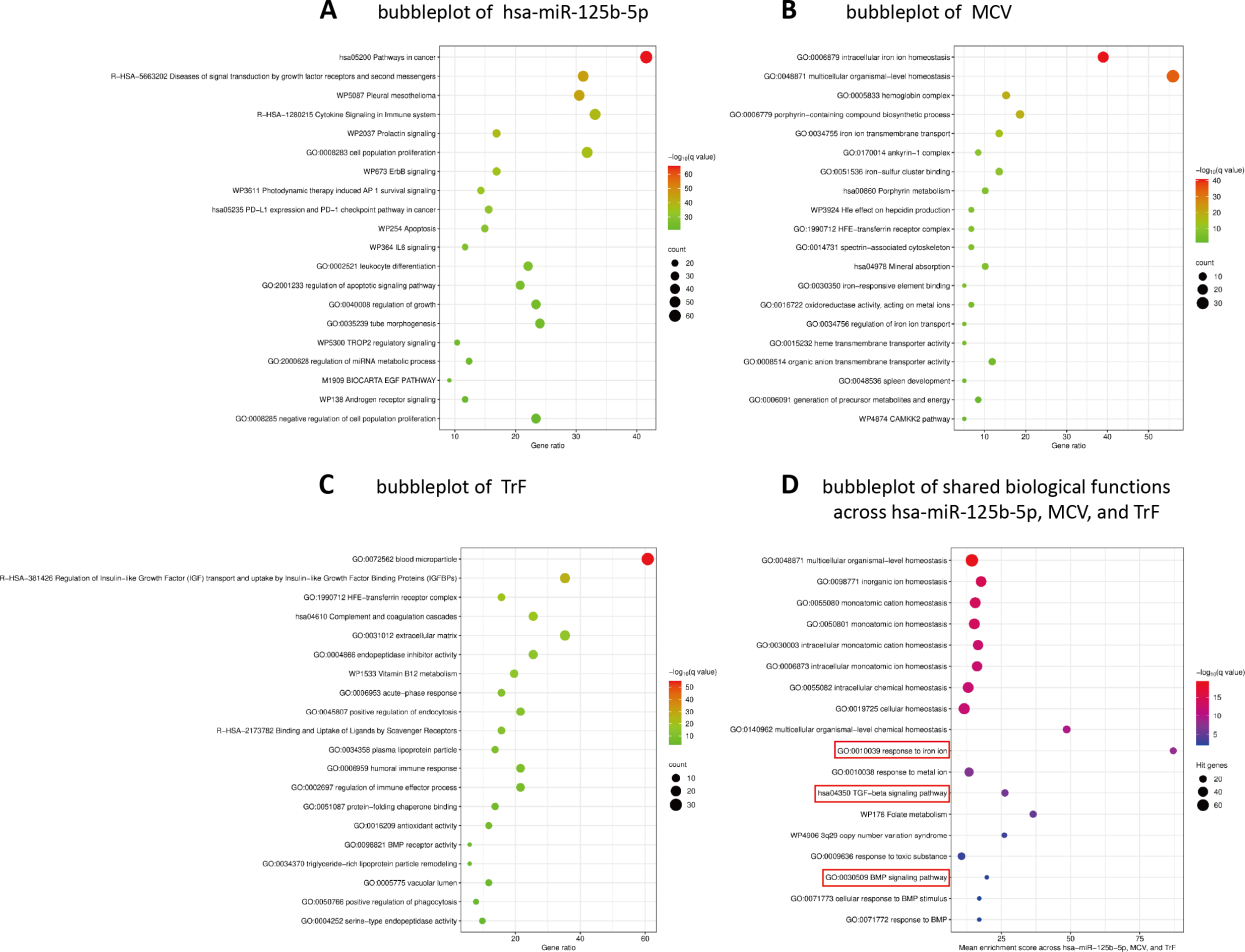
Bubble plots illustrating functional enrichment results of gene networks associated with hsa-miR-125b-5p, MCV, and TrF, as well as their shared biological functions. **(A)** hsa-miR-125b-5p. **(B)** MCV. **(C)** TrF. **(D)** Shared biological functions identified by overlap analysis of enrichment results across hsa-miR-125b-5p, MCV, and TrF, restricted to terms meeting the significance threshold (*q* < 0.05) in all three analyses. In panels **(A–C)**, the x-axis represents the gene ratio, bubble size corresponds to the number of genes involved in each enriched term, and bubble color indicates statistical significance expressed as −log_10_(*q* value), with warmer colors representing stronger enrichment. Only the top 20 clusters with their representative enriched terms (one per cluster) are shown. In panel **(D)**, the x-axis represents the mean enrichment score across the three variables, bubble size corresponds to the number of union genes involved in each enriched term, and bubble color indicates statistical significance expressed as the mean −log_10_(*q* value) across the three variables, with warmer colors representing stronger significance.

Experimentally validated targets of hsa-miR-125b-5p were retrieved from the miRTarBase v8.0 database (https://mirtarbase.cuhk.edu.cn/) (34). Detailed data and additional analyses are provided in **Supplementary Material ["Additional file 3"]**. A total of 458 validated targets were identified, of which 109 targets were supported by strong evidence, including reporter assays, Western blotting, or qPCR. A molecular interaction network was constructed based on these 109 validated targets and their first-neighbor interacting partners. The generated network consisted of 154 nodes and 2854 edges, with network topology centered on TP53, AKT1, MYC, BCL2, STAT3, CTNNB1, PTEN, and CASP3. These genes ranked within the top 10% for both degree and betweenness centrality, indicating their central positions within the network. Detailed data and additional analyses are provided in **Supplementary Material ["Additional file 4", "Additional file 5"]**. Enrichment analysis revealed that the hsa-miR-125b-5p-associated network was significantly enriched in biological functions related to growth factor-mediated signaling, immune and inflammation-associated pathways, and cell fate control mechanisms (**Figure 5A**). Detailed data and additional analyses are provided in **Supplementary Material ["Additional file 6"]**.

#### Targets network and enrichment analysis of MCV

Targets associated with MCV were retrieved from the Human Molecular Signatures Database (MSigDB) v2025.1 (https://www.gsea-msigdb.org/) (35). Detailed data and additional analyses are provided in **Supplementary Material ["Additional file 3"]**. A total of 12 MCV-related targets were identified. A molecular interaction network was subsequently constructed based on these targets and their first-neighbor interacting partners. The generated network consisted of 59 nodes and 520 edges, with network topology centered on ALAS2, FECH, SLC25A37, and TFRC. These genes ranked within the top 10% for both degree and betweenness centrality, indicating their central positions within the network. Detailed data and additional analyses are provided in **Supplementary Material ["Additional file 4", "Additional file 5"]**. Enrichment analysis demonstrated that the MCV-associated network was significantly enriched in biological functions related to iron ion homeostasis, porphyrin and heme metabolism, erythrocyte-associated structural components, and multicellular organismal-level homeostasis (**Figure 5B**). Detailed data and additional analyses are provided in **Supplementary Material ["Additional file 6"]**.

#### Targets network and enrichment analysis of TrF

Targets and first-neighbor interacting partners associated with TrF were retrieved directly from STRING v12.0 by querying the term “transferrin”. A total of 51 genes were identified and used to construct a molecular interaction network. This network consisted of 51 nodes and 720 edges, with TF, ALB, and HP identified as central hub genes based on their ranking within the top 10% for both degree and betweenness centrality. Detailed data and additional analyses are provided in **Supplementary Material ["Additional file 4", "Additional file 5"]**. Functional enrichment analysis identified a coherent set of enriched pathways primarily involving immune and inflammatory processes, complement and coagulation systems, lipid metabolism and particle remodeling, cellular uptake and clearance mechanisms, and growth factor-related signaling (**Figure 5C**). Detailed data and additional analyses are provided in **Supplementary Material ["Additional file 6"]**.

#### Shared biological functions

Overlap analysis was performed on the functional enrichment results of hsa-miR-125b-5p, MCV, and TrF, identifying a total of 35 shared biological functional terms. Detailed data and additional analyses are provided in **Supplementary Material ["Additional file 7"]**. Further filtering was applied to retain terms that met the significance threshold of *q* < 0.05 across all three analyses, resulting in 18 significantly enriched functional terms. The integrated set of 18 functional terms was predominantly associated with biological functions related to cellular and multicellular organismal homeostasis, intracellular ionic and chemical homeostasis, responses to metal ions, and TGF-β/BMP related signaling pathways. These shared enrichment results were visualized using a bubble plot (**Figure 5D**).

## Discussion

Tic disorders are common pediatric neurodevelopmental conditions characterized by substantial clinical and biological heterogeneity (1, 2). Despite increasing evidence implicating genetic, immune, and metabolic factors in their pathophysiology, the molecular regulatory frameworks underlying TD remain incompletely defined (3, 4). MicroRNAs, a class of small non-coding RNAs (36) that regulate gene expression at the post-transcriptional level, have attracted considerable attention as promising biomarkers. Dysregulation of miRNAs has been reported across a wide spectrum of neuropsychiatric disorders, including ADHD (37, 38), OCD (39, 40), anxiety disorders (41), depression (42, 43), autism spectrum disorder (44), bipolar disorder (45), and schizophrenia (46). In recent years, several circulating miRNAs have been proposed as potential biomarkers for TD, highlighting their utility in capturing disease-associated molecular regulatory states. For example, circulating miR-429 has been reported to be significantly downregulated in patients with TS (15). In addition, plasma extracellular vesicle-derived miRNAs, including members of the hsa-let-7 family as well as hsa-miR-25-3p, hsa-miR-29a-3p, hsa-miR-30b-5p, hsa-miR-125b-5p, and hsa-miR-1469, have been shown to discriminate children with TD from healthy controls (16). Certain miRNAs, such as hsa-miR-365b-3p, hsa-miR-485-5p, and hsa-miR-6882-5p, have also been suggested to possess discriminatory potential among different TD subtypes, including PTD, CTD, and TS (16). Moreover, serum hsa-miR-23a-3p has been reported to be upregulated in TS, whereas hsa-miR-130a-3p has been found to be downregulated; differential expression of hsa-miR-21-5p, hsa-miR-25-3p, and hsa-miR-144-3p has also been observed between Arnold-Chiari syndrome (AC) and AC comorbid with TS populations (24). Beyond miRNA dysregulation, TD has been increasingly recognized as a disorder influenced by multiple biological domains, including neurodevelopmental regulation (47–50), immune function (51, 52), genetic susceptibility (3, 53), and metabolic processes (4, 19, 54). Accumulating evidence has suggested a close association between iron metabolism and TD (18, 19), while iron metabolism is tightly coupled with hemoglobin synthesis and erythropoiesis. In addition, alterations in erythroid phenotypes and hemoglobin-related parameters have been proposed to participate in the pathophysiology of psychiatric disorders (23, 55). In this study, an integrative analysis combining circulating miRNAs with erythroid phenotypes and iron-related parameters was conducted to explore peripheral molecular features associated with pediatric tic disorders. Rather than focusing on a single biomarker, our findings highlight coordinated alterations across multiple molecular and physiological domains, suggesting the presence of interconnected regulatory programs relevant to neurodevelopmental pathology.

### Circulating miRNA alterations and interpretation of hsa-miR-125b-5p and hsa-miR-23a-3p signals

Among the examined circulating microRNAs, hsa-miR-125b-5p and hsa-miR-23a-3p were significantly upregulated in children with TD, whereas the remaining candidates exhibited non-significant downward trends. Within the present analytical framework, the observed separation of miRNA expression patterns between groups was largely driven by hsa-miR-125b-5p, indicating its prominent contribution to TD-associated molecular variance. Although hsa-miR-125b-5p did not retain independent significance in multivariate modeling, its inclusion improved the overall explanatory capacity of the integrated feature set, suggesting that circulating miRNAs may provide complementary information within broader regulatory networks rather than acting as isolated indicators.

From a mechanistic perspective, the hsa-miR-125b-5p-associated gene network was centered on canonical hub nodes, including TP53, AKT1, MYC, BCL2, STAT3, CTNNB1, PTEN, and CASP3, and was significantly enriched in growth factor-mediated signaling pathways, immune and inflammatory processes, and cell fate regulation. This pattern is biologically plausible in the context of TD. AKT1 is highly expressed in the prefrontal cortex (PFC) and striatum, and is involved in the regulation of dopaminergic signaling and GABAergic neuronal function in these brain regions (56, 57). Upregulation of the PI3K/Akt signaling pathway has been reported in the striatum of TS model rats, and pharmacological inhibition of PI3K has been shown to attenuate downstream inflammatory signaling while alleviating tic-like behaviors (58). Another study has suggested that PI3K/AKT signaling may influence TD-related phenotypes by modulating microglial activation and the expression of pro-inflammatory mediators (59). STAT3 is mainly expressed in striatal microglia and medium spiny neurons (MSNs), and its activation is closely related to the neuroinflammatory response in the striatum.For example, the JAK2/STAT3 signaling axis may interact with cytokines such as interleukin-6 (IL-6), tumor necrosis factor-α (TNF-α), and interleukin-1β (IL-1β), which have been reported to be associated with TD (51, 60, 61), potentially contributing to neuroinflammatory injury (62). PTEN is highly expressed in the PFC, hippocampus and striatum, and regulates neurogenesis, neuronal migration and synaptic plasticity by antagonizing the PI3K-Akt signaling pathway (63).The PI3K/AKT/mTOR pathway has been implicated in neuronal growth and synaptic remodeling and may contribute to TD pathogenesis (64, 65). TP53, BCL2 and CASP3, the key molecules regulating neuronal apoptosis, are widely expressed in hippocampal neurons, striatal MSNs and cortical pyramidal neurons, and mediate the survival and death of neurons in these brain regions. The TP53-BCL2-CASP3 axis may be relevant to TD through effects on neuronal stress responses, proliferation, or apoptosis (66–70). CTNNB1 (encoding β-catenin) is widely expressed in neurons throughout the brain, and is a core molecule of the Wnt signaling pathway, which is essential for synaptic formation and neurodevelopment (71). MYC is involved in the regulation of neuronal proliferation and differentiation in the developing brain, and is also associated with neuroinflammatory response in the striatum (72).MYC, as a key transcription factor, is involved in the co-expression network of TD-related genes, and may participate in the pathological process of TD by regulating the expression of inflammatory and apoptosis-related genes (73).

The upregulation of hsa-miR-23a-3p observed in the TD group also warrants attention. Functionally, hsa-miR-23a-3p has been implicated in cell fate and survival programs, including effective targeting of PTEN through downstream modulation of the PI3K/Akt signaling pathway (70). In neuronal injury models, hsa-miR-23a-3p has been reported to regulate mitochondrial intrinsic apoptosis involving BCL-2 family members (74). Together, these findings indicate that both hsa-miR-125b-5p and hsa-miR-23a-3p may be involved in convergent signaling pathways relevant to neuronal survival, inflammation, and development. Although the contribution of hsa-miR-23a-3p appeared less dominant than that of miR-125b-5p, the concurrent alteration of hsa-miR-23a-3p suggests coordinated miRNA activity rather than isolated molecular changes.

### Alterations of iron metabolism

Alterations in iron-related parameters and erythroid phenotypes provided additional context for interpreting the observed miRNA-associated molecular patterns. With respect to iron metabolism, SF, TrF, TIBC, and sTfR levels were all reduced in the TD group, whereas serum iron levels did not differ significantly between groups. The reduction in SF observed in children with TD is consistent with previous reports (18). Neuroimaging studies have reported abnormal brain iron content in pediatric patients with TS and have further linked these alterations to tic severity (75). Moreover, convergent evidence integrating neuroimaging and transcriptomic data has supported disturbed iron homeostasis as a pathophysiological feature of TS (19). Collectively, these external findings support the biological relevance of incorporating iron-related phenotypes into a multi-domain biomarker strategy for TD.

Notably, serum iron levels were preserved, whereas TrF, TIBC, and sTfR were lower in the TD group. This distinct iron metabolic profile does not align with the classic pattern of absolute iron deficiency (characterized by reduced serum iron, elevated TrF/TIBC, and increased sTfR), but rather may reflects a state of functional iron deficiency, that a dysregulation of iron transport and cellular utilization despite normal circulating total iron levels. From a physiological perspective, serum iron only represents the total amount of circulating iron, while TrF is the carrier responsible for iron transport in peripheral circulation and across the blood-brain barrier (BBB), with TIBC reflecting the iron-binding capacity of circulating TrF. The synchronous reduction of TrF and TIBC in TD children may indicates impaired systemic iron transport capacity. It is established that brain iron homeostasis is almost entirely dependent on the uptake of circulating TrF-bound iron via transferrin receptor 1 (TfR1) expressed on BBB endothelial cells (76). Therefore, reduced peripheral TrF levels may lead to insufficient iron supply to the key brain regions of the cortico-striatal-thalamic-cortical (CSTC) circuit, which are the core pathological locus of TD. The observed reduction in TrF may also reflect diminished peripheral iron mobilization capacity. The potential upstream mechanism of this iron metabolism disorder may be related to the chronic low-grade inflammatory state. In addition, our study identified that hsa-miR-125b-5p, which was differentially expressed and correlated with iron metabolism indicators in TD children, suggesting that abnormal miRNA expression may also be an important regulatory mechanism underlying the iron transport disorder observed in this study.

Taken together, these findings reveal that the dysregulation of iron transport and cellular utilization, rather than absolute iron deficiency, may be the key pathophysiological feature associated with TD, providing new clues for exploring the pathogenesis and potential intervention targets of this disease.

### Alterations of erythroid phenotypes

At the erythroid phenotype level, Hb concentration and MCV were lower, whereas MCHC was higher in the TD group, while hemoglobin fractions (HbA, HbA2 and HbF) remained unchanged. This combination of lower MCV and mildly reduced Hb does not support overt anemia-driven mechanism. Instead, it suggests more subtle alterations consistent with microcytic tendency and subclinical anemia tendency. Given that erythropoiesis is tightly coupled to iron availability and transport (77), such erythroid phenotypes may represent physiological consequences of altered iron handling that coexist with TD status, rather than disease-specific hematologic abnormalities. Findings from iron metabolism analyses further corroborate this notion. During erythropoiesis, asynchronous iron trafficking and cytoplasmic maturation can give rise to moderately reduced erythrocyte volume, relative more concentrated intracellular hemoglobin, and a trend toward mild insufficiency in total hemoglobin levels. This type of subclinical hematological abnormality may aligns more closely with the systemic subtle homeostatic perturbations associated with neurodevelopmental disorders.

A previous hypothesis proposed that differential hemoglobin fractions may be implicated in neurological or psychiatric disorders, based on the localization of globin genes near genetic loci associated with psychiatric conditions (23). To address this hypothesis, this study conducted a preliminary investigation of hemoglobin components, including HbA, HbA_2_, and HbF. Results revealed no statistically significant differences in these fractions between the TD and NC groups, indicating that the pathogenesis of TD is unlikely to be driven by primary structural hemoglobinopathies. As distinct hemoglobin subtypes arise from variable stoichiometry of α-globin, β-globin, and γ-globin chains encoded by respective globin genes, further speculation suggests that TD may be not significantly associated with aberrant expression or mutations of globin genes.

In addition, the candidate miRNAs in this study are closely implicated in erythropoietic development and maturation, iron metabolic regulation, as well as the initiation and progression of various hematological disorders. Guided by the findings regarding hsa-miR-125b-5p, further mechanistic analysis is performed. Existing studies have confirmed that the miR-125 family mediates stage-specific bidirectional regulation of bone marrow erythropoiesis. In the early stage, miR-125 suppresses the proliferation and differentiation of erythroid progenitor cells by targeting molecules including EPO/EPOR and Lin28A (78), and its dysregulated expression can trigger impaired erythroid maturation and abnormal hemoglobin synthesis, subsequently leading to hematological disorders such as microcytic hypochromic anemia and myelodysplastic syndrome. In the late stage, miR-125 promotes terminal enucleation and maturation of erythrocytes by targeting BCL2 and activating the caspase-3/ROCK1 signaling pathway (79). Concurrently, recent evidence has demonstrated that aberrant expression of the miR-125 family is also involved in the pathophysiological processes of TD. On one hand, hsa-miR-125b-5p has been identified as a potential peripheral biomarker for TD in plasma small extracellular vesicles derived from children (16). On the other hand, dysregulated expression of miR-125a-3p, another member of the miR-125 family, has been detected in brain tissues of rat models of TS (80). Abnormal expression of miR-125 family may affect the function of neural circuits, thereby influencing the condition of children with TD.

Accordingly the candidate miRNAs identified in this study, exemplified by hsa-miR-125b-5p, serve as shared regulatory factors between the central nervous system and the hematopoietic system. Their dysregulated expression may concurrently mediate neural dysfunction, perturbations of erythroid phenotypes and iron homeostasis in TD, thereby acting as a pivotal molecular bridge linking the pathogenesis of TD to peripheral erythroid alterations and systemic iron homeostatic imbalance.

### Convergent pathway signals: homeostasis/metal ion responses and TGF-β/BMP signaling

By integrating the most informative variables, the multivariable model combining hsa-miR-125b-5p, MCV, and TrF demonstrated strong separation capacity at the molecular-feature level. Collectively, the primary value of combined biomarker model (hsa-miR-125b-5p + MCV + TrF) lies in illustrating how molecular and physiological features may jointly capture TD-associated regulatory states. An additional strength of this model lies in its integration of molecular readouts with routinely measured peripheral physiological parameters, which may facilitate conceptual translation across molecular, hematologic, and neurobiological domains. The integration of multimodal models may represent a direction for addressing the clinical rationality of complex disease pathophysiological mechanisms (81–83).

Functional enrichment analyses revealed a certain degree of convergence among hsa-miR-125b-5p, MCV, and TrF across shared biological terms related to cellular and multicellular homeostasis, ionic and chemical homeostasis, responses to metal ions, and TGF-β/BMP-related signaling pathways. This convergence is biologically consistent with systemic iron regulation, in which BMP/SMAD signaling represents a central regulatory axis controlling hepcidin expression and iron homeostasis (84–86). In parallel, signaling pathways belonging to the TGF-β/BMP superfamily are known to participate in neuronal development, maintenance of neuronal activity, and synaptic regulation, and dysregulation of these pathways has been implicated in a range of neurological and neuropsychiatric disorders (87). Although circulating hepcidin levels were not assessed in the present study, the observed convergence of enrichment results supports the interpretation that the integrated biomarker set may reflect upstream regulatory programs, rather than isolated alterations of individual analytes.

Notably, in order to further integrate the targets and biological functions shared by the three variables (hsa-miR-125b-5p, MCV, and TrF), several genes (BMPR1B, HFE, HJV, TFR2, and TFRC) were repeatedly identified among the hit genes associated with shared biological terms, involving in at least two variables and two biological terms. These genes are prominently involved in iron homeostasis.

HFE, TFRC, HJV, and TFR2 are core regulators of systemic iron homeostasis. TFRC (transferrin receptor) mediates cellular iron uptake, which is essential for erythropoiesis and iron delivery to tissues (76, 88). HJV (hemojuvelin) acts as a co-receptor to enhance BMP/SMAD signaling, a central axis regulating hepcidin expression and iron homeostasis (89). TFR2 (transferrin receptor 2) is mainly expressed in hepatocytes, where it binds to transferrin to facilitate iron entry and regulates hepcidin levels (90). HFE (hemochromatosis gene) forms a complex with TFR2 to regulate hepcidin synthesis, and its dysfunction disrupts systemic iron balance (90). As shared hit genes of MCV and TrF, these four genes directly link iron transport to erythropoietic function. A hypothetical directional association was proposed, that HFE, TFRC, HJV, and TFR2 may impair iron sensing and transport functions, reduce the utilization efficiency of iron in erythropoiesis, and lead to decreased MCV and abnormal TrF levels in TD patients.

The primary mechanism in systemic iron homeostasis is the regulation of hepcidin expression by coordinated membrane sensing and BMP-SMAD signaling. Within this framework, BMPR1B (bone morphogenetic protein receptor 1B) and other BMP receptors functionally integrate with the upstream HFE/TFR2/HJV complex to regulate the BMP-SMAD-hepcidin axis, linking extracellular iron sensing to hepatic iron regulatory responses (91, 92). BMPR1B may act as a functional link, connecting miR-125b-5p dysregulation to peripheral iron transport and central signaling pathways relevant to TD. A hypothesis is proposed that hsa-miR-125b-5p may influence iron homeostasis and neural developmental signaling mediated by the BMP/SMAD pathway by regulating BMPR1B.

Notably, the upregulation of hsa-miR-125b-5p observed in the TD group may have dual biological implications. It may exert pathogenic effects by inhibiting target proteins expression, or as a compensatory protective response to alleviate TD-related pathological damage. On the one hand, miR-125b-5p upregulation may exert pathological effects by suppressing target genes involved in neuroprotection and iron homeostasis. Overexpression of miR-125b has been shown to induce tau hyperphosphorylation and cognitive deficits in Alzheimer’s disease by downregulating anti-apoptotic factors and phosphatases (93, 94). It also promotes microglial M1 polarization and neuroinflammation in spinal cord injury, exacerbating neuronal apoptosis (95). On the other hand, miR-125b-5p upregulation may represent a compensatory protective mechanism to counteract TD-related pathological stress. Studies have shown that miR-125b mimic can inhibit ischemia-reperfusion-induced neuroinflammation and aberrant p53 apoptotic signaling by targeting TP53INP1, thereby protecting neurons and improving motor function *in vivo* (96).

**Figure 6:**
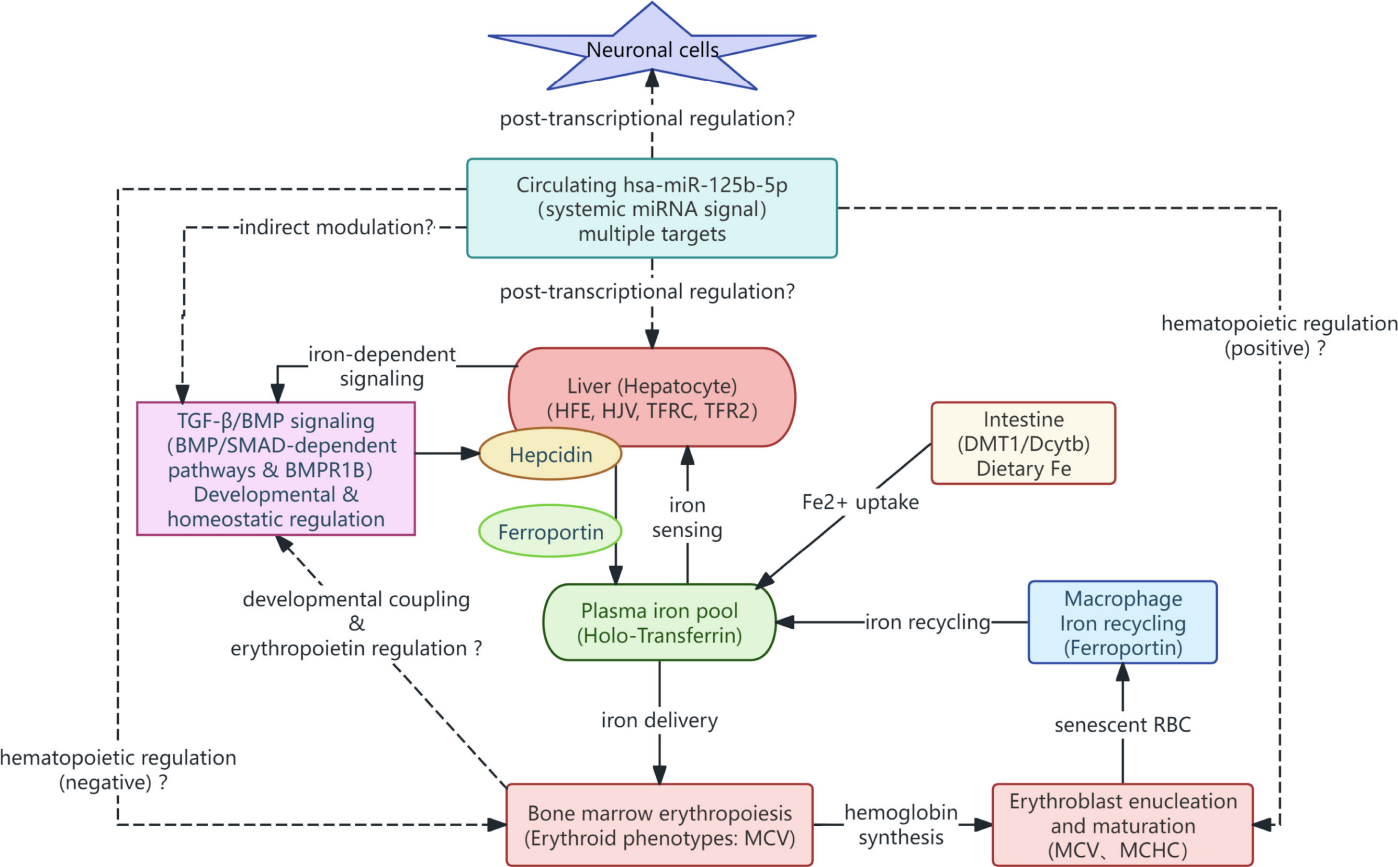
Mechanistic hypothesis linking hsa-miR-125b-5p, iron homeostasis and erythroid phenotype. The solid lines represent known mechanism directions, while dashed lines indicate unknown speculative directions. It involves neuronal cells, liver, TGF-β/BMP signaling, hepcidin, Ferroportin, iron pool, macrophages, bone marrow erythropoiesis, and erythrocyte maturation, including feedback mechanisms and iron-dependent signaling.

Collectively, these hit genes form interconnected modules that converge on pathways related to homeostasis, metal ion regulation, and TGF-β/BMP signaling (**Figure 6**). The potential dual role of miR-125b-5p adds complexity to the molecular mechanisms underlying TD, suggesting that further experimental studies are needed to clarify its specific functional role.

### Limitations and future perspectives

Several limitations should be acknowledged. First, this study was designed as an exploratory investigation for biomarker screening and hypothesis generation, and the sample size was determined with reference to previous exploratory circulating miRNA studies in pediatric TD (16). The relatively small sample size may lead to limited statistical power and generalizability. Accordingly, the present findings should be regarded as preliminary trends rather than definitive conclusions. Future validation in larger sample, independent, multi-center external cohorts using consistent laboratory protocols, enrollment criteria, detection platforms, and statistical models is required to confirm the stability and reproducibility of the identified miRNAs and combined biomarker panel. Secondly, this study adopted a cross-sectional observational study, which limits causal inference and does not allow definitive conclusions regarding whether the observed molecular alterations precede disease onset or arise as a consequence of the disorder. The observed changes should be interpreted as co-occurring biological characteristics rather than drivers or causal mechanisms of TD. Thirdly, the diagnostic model established in this study was evaluated solely through internal cross-validation and bootstrap methods, and currently lacks external validation in independent cohorts, which may pose a risk of overfitting. This represents the primary limiting factor restricting the clinical translation and practical application of the model. Future validation in large-scale, multicenter independent cohorts is required before considering clinical application. Although comorbidities have been ruled out, unassessed subclinical symptoms may still exist, and future studies could incorporate stratification based on comorbidities. In addition, peripheral molecular and hematologic measurements may not directly reflect central nervous system processes. Circulating miRNA levels cannot be equated with intracellular miRNA levels, and their regulatory mechanisms require further exploration through animal experiments or *in vitro* studies. Nevertheless, the present findings support the feasibility of integrative, network-based approaches for exploring molecular regulation in pediatric TD.

## Methods

### Ethics statement

This study was conducted at Guang’anmen Hospital, China Academy of Chinese Medical Sciences. The study protocol was reviewed and approved by the Ethics Committee of Guang’anmen Hospital. Written informed consent was obtained from the parents or legal guardians of all participants prior to enrollment and blood sample collection.

### Participants

A total of 30 children diagnosed with tic disorders [TD group; age range 5-16.8 y; mean age 8.93 y (SD ± 2.80); sex M:F = 24:6] and 10 children undergoing routine physical examinations as normal controls [NC group; age range 7-18 y; mean age 10.25 y (SD ± 3.16); sex M:F = 8:2] were recruited.

All TD participants were clinically diagnosed by qualified pediatric clinicians according to the DSM-5-TR (1). Normal controls had no history of neuropsychiatric disorders based on medical record review and routine clinical evaluation. To minimize potential confounding effects, only children with TD without other neurodevelopmental or psychiatric comorbidities were enrolled, and none had active infections or acute inflammatory conditions at the time of blood sampling.

At enrollment, all participants were confirmed to have not received any medications for TD, psychiatric conditions, or agents that may affect hematological parameters for at least 1 month prior to study entry, thereby excluding potential residual effects of previous treatments. In addition, participants were excluded if they had received iron supplements, folic acid, hematinic agents, blood transfusion, or had been diagnosed with thalassemia, polycythemia, or other hematological diseases that may influence erythrocyte-related indicators.

### Blood collection and processing

Fasting peripheral venous blood was collected by experienced nurses following standard clinical procedures in the morning. For high-resolution hemoglobin component analysis, 2 mL of peripheral blood was collected into EDTA anticoagulant tubes, stored at 4 °C, and transported to Beijing KingMed Clinical Laboratory. Hemoglobin fractions (HbA, HbA_2_, and HbF) and erythroid phenotypes, including RBC, Hb, MCV, MCH, and MCHC, were measured using automated hematology analyzers and hemoglobin electrophoresis systems.

For circulating miRNA quantification and iron metabolism testing, 3 mL of peripheral blood was collected into serum separation tubes containing clot activator and gel. Serum separation was performed by the Clinical Laboratory of the hospital. A 200 µL serum aliquot was used for iron metabolism assessment, including SF, TrF, SI, sTfR, and TIBC, measured on an automated analyzer in the Clinical Laboratory of the hospital. An additional 200 µL serum aliquot was transferred into a 1.5 mL RNase-free Eppendorf tube for circulating miRNA assessment. The serum samples were stored at -70 °C until further use.

### Circulating miRNA extraction and RT-qPCR

Total RNA was extracted from 200 µL of serum using the miRcute Serum/Plasma miRNA Isolation Kit (DP503, TIANGEN) according to the manufacturer’s instructions. RNA was eluted in a total volume of 30 µL RNase-free ddH_2_O using two consecutive elution steps and stored at -70 °C until reverse transcription.

MiRNA-specific reverse transcription was performed using the miRcute Plus miRNA First-Strand cDNA Kit (KR211, TIANGEN). The resulting cDNA was stored at -20 °C until quantitative PCR analysis. Quantitative PCR was conducted using the miRcute Plus miRNA qPCR Kit (SYBR Green, FP411, TIANGEN) in a 20 µL reaction volume on a LightCycler^®^ 480 Real-Time PCR System (Roche). MiRNA-specific forward primers were provided by TIANGEN (CD-201 series).

### Candidate miRNA identification

A two-source strategy was applied to identify TD-related miRNAs and prioritize those also implicated in erythroid/hemoglobin-related disease contexts. HMDD v4.0 (28) was queried to retrieve miRNAs associated with TDs and erythroid/hemoglobin-related diseases, which was a curated resource of experimentally supported miRNA-disease associations. In parallel, disease-related expression data were retrieved from the GEO database and published literature (15, 24, 29, 30), and miRNAs reported as differentially expressed in both disease categories were supplemented. MiRNAs supported by both disease annotation and expression evidence were retained as candidate miRNAs. Overlap analysis was visualized using EVenn (https://www.bic.ac.cn/test/venn/#/) (97).

### Targets retrieval and functional enrichment analysis

Targets and first-neighbor interacting partners associated with key variables were retrieved using the STRING v12.0 database (32), the miRTarBase v8.0 database (34), and MSigDB v2025.1 (35). Gene interaction networks were constructed and analyzed using Cytoscape v3.8.1. Functional enrichment analysis was performed using Metascape v3.5.20250701 (33), comparing network genes against the whole-genome background. Enrichment analyses were conducted across multiple databases, including GO, KEGG, Reactome, WikiPathways, and BioCarta. Statistical significance was assessed using hypergeometric testing with Benjamini-Hochberg false discovery rate correction.

### Statistical analysis

Statistical analyses were performed using R 4.5.2. Continuous variables were summarized as mean ± standard deviation (SD). Group comparisons between TD and NC groups were conducted using independent-samples *t*-tests for variables with normal distribution and homogeneous variance, and *Mann-Whitney U* tests for otherwise. Pearson correlation analysis was used for continuous variables with normal distribution, otherwise Spearman rank correlation analysis was applied. For subgroup comparisons, *one-way ANOVA* or *Kruskal-Wallis H* test was used, followed by *post hoc* multiple comparisons with correction for pairwise comparisons between groups. Statistical significance was defined as *p* < 0.05. Relative miRNA expression levels were calculated using the 2^-ΔΔCt^ method with hsa-U6 as the endogenous reference and the NC group as the calibrator (98, 99). For visualization, log_2_ fold change values (log_2_FC) were z-score-standardized prior to heatmap construction. This transformation was applied for visualization purposes only. Diagnostic performance was evaluated using logistic regression models and ROC curve analysis, reporting AUC values with 95% CI. Internal validation was performed using cross-validation and bootstrap resampling. Figures were generated using GraphPad Prism v10.1.2, Adobe Illustrator 2025, and the SRplot online platform (https://www.bioinformatics.com.cn; last accessed on 28 Dec 2025) (100).

## Data Availability

The original contributions presented in the study are included in the article/**Supplementary Material**, further inquiries can be directed to the corresponding author/s.
